# Radiomics and Dosiomics for Predicting Local Control after Carbon-Ion Radiotherapy in Skull-Base Chordoma

**DOI:** 10.3390/cancers13020339

**Published:** 2021-01-18

**Authors:** Giulia Buizza, Chiara Paganelli, Emma D’Ippolito, Giulia Fontana, Silvia Molinelli, Lorenzo Preda, Giulia Riva, Alberto Iannalfi, Francesca Valvo, Ester Orlandi, Guido Baroni

**Affiliations:** 1 Department of Electronics, Information and Bioengineering, Politecnico di Milano, Piazza Leonardo da Vinci 32, 20133 Milan, Italy; chiara.paganelli@polimi.it (C.P.); guido.baroni@polimi.it (G.B.); 2 Radiotherapists Unit, National Center of Oncological Hadrontherapy (CNAO), Strada Campeggi, 53, 27100 Pavia, Italy; emma.dippolito@gmail.com (E.D.); giulia.riva@cnao.it (G.R.); alberto.iannalfi@cnao.it (A.I.); francesca.valvo@cnao.it (F.V.); ester.orlandi@cnao.it (E.O.); 3 Clinical Bioengineering Unit, National Center of Oncological Hadrontherapy (CNAO), Strada Campeggi, 53, 27100 Pavia, Italy; giulia.fontana@cnao.it; 4 Medical Physics Unit, National Center of Oncological Hadrontherapy (CNAO), Strada Campeggi, 53, 27100 Pavia, Italy; silvia.molinelli@cnao.it; 5 Radiology Unit, National Center of Oncological Hadrontherapy (CNAO), Strada Campeggi, 53, 27100 Pavia, Italy; lorenzo.preda@cnao.it; 6 Unit of Radiology, Department of Intensive Medicine, IRCCS Policlinico San Matteo, 27100 Pavia, Italy

**Keywords:** radiomics, dosiomics, machine learning, particle therapy, oncology, radiology, personalized medicine

## Abstract

**Simple Summary:**

Skull-base chordomas (SBC) are rare tumours with unfavourable outcomes, even when undergoing advanced treatments such as carbon-ion radiotherapy (CIRT). By retrospectively analysing imaging (MRI, CT), treatment (dose maps) and clinical information available before treatment, the potential use of radiomics and dosiomics for risk modelling targeting SBC treated with CIRT was explored. Despite the small sample size, dosiomic features appear to be promising factors related to local control in SBC, with worse outcomes being associated to higher dose heterogeneity. Risk models exploiting all sources of information showed slightly inferior but good performance, suggesting that multi-parametric approaches are worth being pursued for patient risk stratification. This study is put forward as groundwork for radiomic analyses targeting SBC in CIRT.

**Abstract:**

Skull-base chordoma (SBC) can be treated with carbon ion radiotherapy (CIRT) to improve local control (LC). The study aimed to explore the role of multi-parametric radiomic, dosiomic and clinical features as prognostic factors for LC in SBC patients undergoing CIRT. Before CIRT, 57 patients underwent MR and CT imaging, from which tumour contours and dose maps were obtained. MRI and CT-based radiomic, and dosiomic features were selected and fed to two survival models, singularly or by combining them with clinical factors. Adverse LC was given by in-field recurrence or tumour progression. The dataset was split in development and test sets and the models’ performance evaluated using the concordance index (C-index). Patients were then assigned a low- or high-risk score. Survival curves were estimated, and risk groups compared through log-rank tests (after Bonferroni correction α = 0.0083). The best performing models were built on features describing tumour shape and dosiomic heterogeneity (median/interquartile range validation C-index: 0.80/024 and 0.79/0.26), followed by combined (0.73/0.30 and 0.75/0.27) and CT-based models (0.77/0.24 and 0.64/0.28). Dosiomic and combined models could consistently stratify patients in two significantly different groups. Dosiomic and multi-parametric radiomic features showed to be promising prognostic factors for LC in SBC treated with CIRT.

## 1. Introduction

Particle therapy makes use of charged particles such as protons or carbon ions and is increasingly being adopted worldwide, with over 50 facilities built in the last ten years [1]. Although carbon ion radiotherapy (CIRT) is limited to specialized centres, it shows higher geometrical selectivity and increased radiobiological effectiveness with respect to proton and conventional X-ray radiotherapy, thus being indicated for treating radioresistant and deep-seated tumours [2], such as chordomas.

Skull-base chordoma (SBC) is a rare but aggressive tumour, locally invasive and highly recurrent [3]. Given the anatomical location, the combination of surgery and particle therapy [4] is suggested for treatment [5], but tumour response remains not satisfactory (5-year survival rate: 45% for conventional radiotherapy, 87% for CIRT [6]; 5-year local control: 48–60% [5] and 72% [7], respectively). Additionally, the limited phenotypic characterization of SBC prevents an optimal patient stratification to improve treatment outcomes. In this context, the growing availability of imaging data can be favourably exploited as a source of prognostic factors [8,9], with studies in the literature supporting the predictive power of the appearance of chordomas on diagnostic imaging, such as CT and MRI [10]. More recently, qualitative imaging factors are being complemented by quantitative ones, such as radiomic features [11].

Radiomics refers to the automatic extraction of quantitative imaging features, which can be divided in shape, first order, textural and filter-based features, according to the specific characteristic described, to develop predictive models [12]. The general hypothesis of radiomics is that imaging characteristics reflect physiopathological tissue information, which is thus made accessible through quantitative features [13]. It is then reasonable to assume that different imaging contrasts or modalities describe complementary characteristics and that multi-parametric approaches can be beneficial for predictive tasks [14]. Among image modalities, CT [15,16] and PET [17,18] were the focus of several radiomics studies, as they are the most widely adopted and standardized imaging modalities in radiotherapy workflows. Even if MRI exhibits a greater variability in acquisition protocols that hinders the collection of large datasets [19], a growing interest in MRI-based radiomic features in neuro-oncology is observed in the literature [20,21,22]. In addition, in conventional X-ray radiotherapy studies [23,24,25], the extraction of radiomic features from dose maps (i.e., dosiomics) have been proposed, so that the delivered treatment can be characterized by descriptors of spatial patterns in dose distributions, against the conventional point-wise parameters of dose-volume histograms (DVH). Finally, the combination of multiple types of potential prognostic factors, from radiomic to clinical features, was also suggested to improve the performance of predictive models [26].

Despite its potential clinical usefulness, the radiomic paradigm has been applied to few studies targeting SBC, mostly focused on diagnostic tasks [27]. Promising results in predicting local recurrences were obtained from wavelet features extracted from contrast-enhanced MRI on surgically-treated clival chordomas [28]. To date, however, no radiomic model on SBC response to CIRT has been developed but, according to the radiomic concept, it may be a valuable support to clinical decisions in the radiation therapy workflow.

The aim of this study was to explore radiomic approaches for predicting local control in SBC treated with CIRT. Towards this goal, radiomic and dosiomic features were extracted from routinely acquired pre-treatment imaging and dose maps, which were selected, combined, enriched with clinical information, and fed to time-to-event models. Such framework is, therefore, put forward as the groundwork towards the identification of the most promising radiomic (and dosiomic) workflow for deriving prognostic factors of SBC response to CIRT.

## 2. Results

### 2.1. Patient Data

Imaging (T1w-MRI, T2w-MRI, CT), treatment (dose maps) and clinical data was retrospectively collected for 57 SBC patients treated with CIRT. Local control (LC), i.e., favourable outcome, was found in 70% of the patients after a median follow-up time of 35.2 months (range: 2.9–66.07 months). Clinical features (Table 1) were recorded according to clinical practice. Missing values from categorical variables (marked as n.a. in Table 1) were replaced by the mode of each features’ distribution.

Survival models were developed exploiting 80% of the dataset (*n* = 45) within a cross-validation procedure and further tested on the remaining 20% (*n* = 12) of the data, to evaluate the models on totally unseen samples. Data was split randomly but ensuring that the proportion of samples associated to adverse and favourable LC was equal (71% vs. 66% positive LC for model development and hold-out test, respectively).

### 2.2. Single Modality

At first, the extracted features were fed to survival models, separately, to investigate the capability of each single modality to provide prognostic features. Different feature selection routines were evaluated, along with two survival models (i.e., linear survival support vector machines, s-SVM, vs. Cox proportional hazards model regularized with an elastic net penalty, r-Cox). Performance was assessed in terms of the concordance index (C-index) computed from a stratified five-fold cross-validation (CV) routine over the training set (validation C-index) for model development and over the hold-out test set (test C-index).

**Figure 1:**
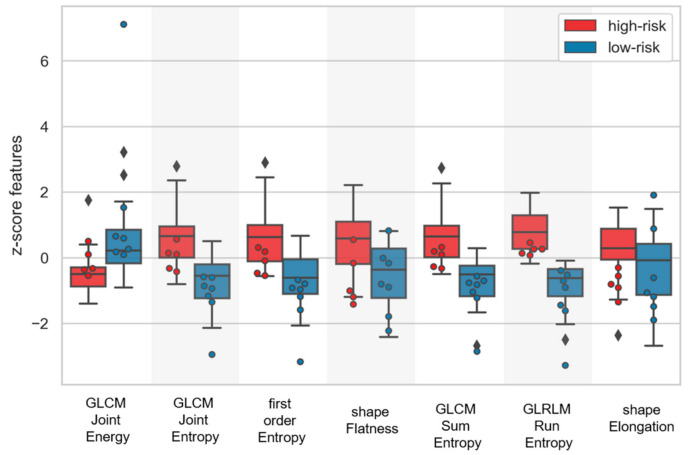
Standardized dose features for patients as stratified by the best performing r-Cox model (i.e., highest validation C-index) according to the risk (high in red, low in blue) of showing an adverse local control. The model was re-trained on the whole training set (80% dataset), from which the stratification cut-off was estimated, and tested on the hold-out test set (20% dataset). Boxplots refer to re-training data, whereas the overlaid points refer to test data.

The signatures (supplementary materials, Section S5) associated to the best models (i.e., highest validation C-index) in single-modality cases consisted of different features types, as described in the following:

- Selected T1w-MRI and T2w-MRI features (supplementary materials, Figures S1 and S2) belonged to the groups of first order, textural and shape features.
- Selected CT features (supplementary materials, Figure S3) described various image properties, such as the distribution of low and high HU values (first order 10th percentile and GLRLM High Gray Level Run Emphasis, HGLRE), or their variability (e.g., first order robust Mean Absolute Deviation, rMAD). Additionally, regional (e.g., GLSZM Large Area Emphasis) and volume-confounded descriptors (e.g., first order Energy) were selected.
- Selected dosiomic features (Figure 1, supplementary materials, Figure S4) mostly described heterogeneity at different spatial scales (GLRLM run entropy, RE; GLCM Joint Energy, JEg; GLCM Joint Entropy, JEp; GLCM sum entropy; first-order entropy) and shape properties (elongation, flatness).

Overall, r-Cox performed better than s-SVM (Table 2, supplementary materials Table S4), being the validation C-indices above randomness in most of the single-modality cases (87% vs. 71% of cases for r-Cox and s-SVM). MRI-based s-SVM showed the worst results, with validation C-indices exceeding 0.50 in only 50% of the cases (80% for r-Cox). However, s-SVM was able to achieve slightly higher peak performances. The overall best performance was achieved by dosiomic models both for s-SVM (validation C-index as median/interquartile range: 0.80/0.24) and r-Cox (0.79/0.26), followed by CT-based ones (0.77/0.24 for s-SVM; 0.64/0.28 for r-Cox). The clinical signature led to sufficient validation C-indices (0.69/0.23 for s-SVM, 0.64/0.26 for r-Cox).

### 2.3. Combined Modalities

Following single modality analyses, imaging, dose and clinical information with the best validation C-indices were combined to evaluate the performance of a multi-parametric scenario. 

In the best-performing cases of comboAll models, the correlation-based feature selection method favoured clinical and dosiomic features, whereas the PCA-based method retained features from all modalities (supplementary materials, Figure S5). Textural dosiomic (GLRLM RE) and shape (flatness) features were found in all signatures fed to the best comboAll r-Cox models. 

Regarding models’ performance, the best r-Cox performed slightly better than the best s-SVM (0.75/0.28 vs. 0.73/0.30), but they did not outperform the best single-modality dosiomic models.

### 2.4. Survival Analysis

**Figure 2:**
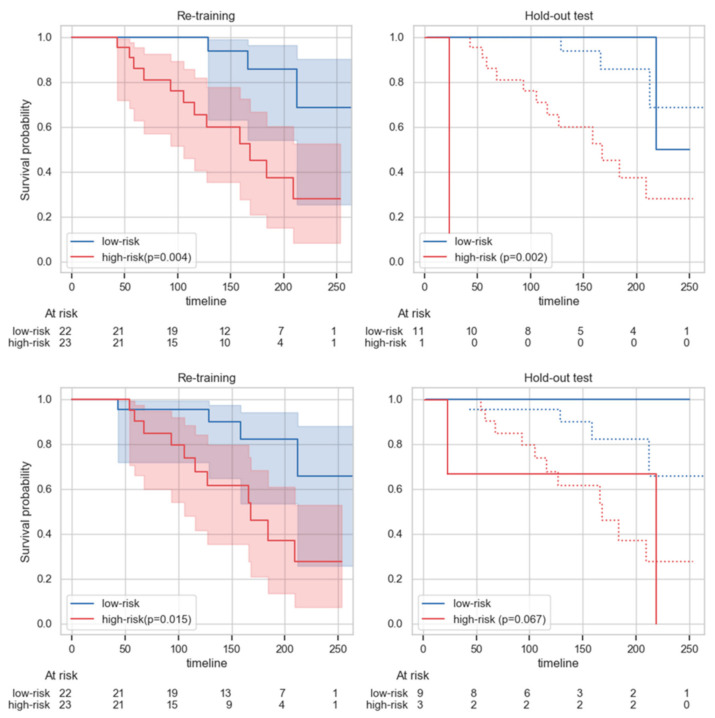
Kaplan Meier survival curves for patients at high-(red) and low-risk (blue) of meeting an adverse local control as stratified by s-SVM models for the best dose case (top) and comboAll (bottom), after re-training. An estimator is fit on the re-training data (left, shaded areas depict curves’ confidence intervals) and applied to the test data (continuous lines on the right; dashed lines represent the re-training curves shown on the left). Below each plot, the number of patients belonging to each risk group at certain times is shown. The *p*-values (p) in the legends refer to the comparison of high- and low-risk patient groups within re-training (left) or test (right) set using log-rank tests.

The survival curves (Figure 2) of high- and low-risk hold-out test patients, as defined according to models’ output, significantly differed (log-rank test, α = 0.0083) only for the dosiomic s-SVM model (Table 3). Results from re-training data, even if over-optimistic, show significant differences in T2w-MRI, CT, dose, comboAll and clinical models, only for s-SVM. All the models tested on the hold-out test set showed optimal test C-indices (supplementary materials, Section S6, Table S5), apart from MRI-based ones.

## 3. Discussion

In this study, survival models were investigated to stratify SBC patients treated with CIRT according to the risk of an adverse local control. Information available before treatment was exploited, including radiomic features extracted from T1w-MRI, T2w-MRI and CT, dosiomic features and clinical factors recorded according to the clinical practice.

### 3.1. Technical Evaluation

By exploring various feature selection routines, it was possible to investigate different feature signatures while mitigating the mismatch between number of features and sample size.

To further account for the problem of limited sample size, s-SVM was the chosen machine learning model, as it is relatively robust to overfitting [30], and it was compared to a traditional statistical model (r-Cox). As supported by the literature [31], no unique combination of a feature selection method and a model outperformed the other combinations across all input feature types. Both models provide linear decision boundaries, but the possibility to tune the step size of r-Cox (supplementary materials, Section S3) may explain its slightly higher average prognostic performance and stratification capabilities, as suggested by the C-index and the long-rank tests, respectively. In this study, non-linear models (e.g., kernel s-SVM) were not investigated, since a higher risk of overfitting is associated to an increased model’s complexity [13]. Nevertheless, these models would certainly be of interest if more data samples were available. 

Additionally, the limited test set available for the current study hinders the evaluation of the test C-index alone, which often reached its maximum value. However, this over-optimism is expected to fade once the hold-out test data is expanded.

### 3.2. Single Modality

The MRI features that led to the best MRI-based models described shape, histogram and textural properties (supplementary materials, Figures S1 and S2), but were of limited generalizability, especially for T1w-MRI as shown by the C-indices and *p*-values obtained on the hold-out test set. Such behaviour may be explained by different factors. Firstly, MR-radiomic features were extracted from gross tumour volumes (GTV) that had been delineated on a fused MR-CT and rigidly registered to MRIs, as no direct manual contour was available for each MR modality. As such, the contour may not exactly match the MR-visible tumour due to registration errors. Moreover, MRI were retrospectively collected and, even if most of the acquisition parameters were matched, some of them (e.g., echo time) varied more if compared to acquisition and computation parameters of CT and dose maps, respectively. This and the lack of test-retest data may cause non-reproducible features to be fed to the models [19,32] which are not able to generalize to unseen data. Due to the limited data available, it was not possible to separately investigate the potential confounding factors (contouring, intrinsic features reproducibility, variations in acquisition parameters) which are known to affect the computation of radiomic features [33]. Nonetheless, given the radiological relevance of chordoma appearance on MRI [11], from which haemorrhage, calcifications and other heterogeneous structures can be identified, quantitative (e.g., diffusion-weighted MRI) and standardized anatomical MR sequences (e.g., fat-saturated or contrast-enhanced) should be explored as promising sources of prognostic features [27,34,35]. Indeed, textural wavelet features from anatomical T1w- and T2w-MRI showed to be promising for SBC treated with surgery [28]. In the current study, based on patients who already underwent surgery and enrolled for CIRT, features from wavelet-filtered image were not analysed to reduce the risk of overfitting. Nevertheless, it would be interesting to explore wavelet MRI features for SBC treated with CIRT, once a larger and more homogeneous MR dataset is gathered.

Although CT offers a lower soft tissue contrast than MRI, the selected CT features (supplementary materials, Figure S3) showed comparable or higher validation C-indices with respect to MRI. This could be due to the tendency of chordomas to segregate, infiltrate and destroy bone structures [11], which are well identifiable on CT imaging. This observation seems to be supported by the choice of features pertaining low (10th percentile) or high (GLRLM HGLRE) or differences between low and high (rMAD) HU values, in the best performing cases. Overall, shape features also contributed to the performance of many best models, suggesting that GTV geometry descriptors could play a role.

Dosiomic features turned out to be the most promising signatures (Table 2), as shown by the validation C-indices, which comprised shape and dose textural features in the best cases. This agrees with literature findings as the presence of low-dose regions and dose inhomogeneities within the GTV is one of the primary causes of local recurrences [36]. All textural features, apart from GLCM JEg, appeared to be higher for patients showing an adverse LC, who were thus described by lower homogeneity (GLCM JEp) and higher heterogeneity (GLRLM RE, GLCM JEp and SE) in the planned biological dose (Figure 1, supplementary materials Figure S4). First-order dosiomic features resembled dose-volume histogram (DVH) indices but, apart from entropy, which still measures heterogeneity, no first-order feature was selected in the signatures associated to the best cases. This suggests that dose spatial patterns may have a higher impact on the success of CIRT treatments with respect to conventional DVH metrics and future studies should focus on their rigorous comparison [23,36]. The improved performance of dosiomic with respect to radiomic models could be explained by the higher standardization of the dose protocol for this patient cohort. However, since biological dose maps were employed to account for CIRT biological effects, a generalization of these results to other radiation treatments (e.g., proton and X-ray) cannot be directly made and should be carefully evaluated [37]. Even within CIRT doses, it would be interesting to compare these findings with those coming from different radiobiological models, which are known to strongly affect RBE calculations [38,39]. 

Models based on clinical features did not outperform dosiomic models but showed comparable results to radiomic models. Clinical variables were limited to those available for most of the patients but other factors, missing from the current evaluation (e.g., extent of surgical resection), may be beneficial and their impact of LC in SBC treated with CIRT should be investigated [8]. Clinical models may be more easily generalizable to other treatment modalities with respect to dosiomic and radiomic models, but they may be subject to patient selection biases. If clinical and demographic characteristics of patients eligible for CIRT differ from those of patients undergoing X-ray or proton treatments [36], care must be paid also when generalizing clinical models to other therapeutic strategies. 

### 3.3. Combined Modalities

When combining all sources of information, features leading to the best models within each group (i.e., MRI, CT, dose, clinical) were merged, selected, and evaluated (comboAll). The best validation C-indices slightly lowered with respect to those from dosiomic models (0.73 vs. 0.80 for s-SVM; 0.75 vs. 0.79 for r-Cox) but improved with respect to those from radiomic and clinical models. In the best comboAll cases, the selected clinical features were anatomical location, optic pathway involvement, and/or gender. This agrees with recent studies [8,36] that investigated the prognostic power of clinical factors and showed a consistent association to worse outcomes when optic pathways were affected (clinical visual deficits or radiological involvement), which may be related to the impact of the constraints for critical structures on the prescribed dose. The beneficial impact of dosiomic features was confirmed by the subset of features that led to the best r-Cox model (five dosiomic features out of the 10 selected) and by the consistent choice of GLRLM RE in all best comboAll models (supplementary materials, Figure S5). Finally, radiomic features from MRI also contributed to build best-performing models in comboAll, thus (i) supporting the importance of considering different sources of information and (ii) indicating that multi-modal approaches could potentially mitigate shortcomings related to single modalities.

### 3.4. Validity and Limitations of the Proposed Work

The clinical usefulness of dosiomic and comboAll models is supported by the separation of survival curves obtained for low- and high-risk groups. Only the dosiomic s-SVM model could significantly separate low- and high-risk patients in both re-training and hold-out test sets. However, the significant results observed in the re-training set, although over-optimistic, suggest that statistical significance may be found in larger hold-out test sets when using s-SVM models. As only a single stratification cut-off (median value) was evaluated and these results should be considered cautiously [31], this analysis is put forward as an example of the clinical usefulness of the proposed method.

Moreover, given the relatively small sample size and the mono-centric and retrospective study design, the reported results need to be validated by broader analyses, considering additional data either coming from the same or an external institution [40]. In the latter case, advanced harmonization techniques should be considered, especially for anatomical imaging [41] and biological doses [38]. As an external validation is of paramount importance to correctly evaluate the generalization capabilities of the proposed framework [42], future work will focus on extending the current study to data coming from other institutions. Additionally, even if GTVs were delineated following institutional guidelines, future efforts will be put in quantifying inter-observer variabilities and evaluating the feasibility of automatic segmentation strategies for SBC [43]. Whereas delineation variabilities are known to affect radiomic features at various degrees [19], no reproducibility study has been conducted on dosiomic features yet. This can be explained by the difficulties arising from the influence that contours have on both the dosiomic features and the planned doses, from which features are then computed. Studies aiming at evaluating the impact of contouring on dosiomic features must be carefully planned to be able to address such dependency.

Other than technical aspects, clinical and biological validations need to be addressed before radiomic and dosiomic features can be employed as biomarkers for SBC [44]. This is even more relevant in the case of CIRT, for which the radiobiological effectiveness is one of the major benefits with respect to conventional radiotherapy. Finally, it should be also considered that the relatively small sample available for this study represents a unique dataset coming from a peculiar treatment, such as CIRT, that is often reserved to rare tumours.

## 4. Materials and Methods

### 4.1. Patient Data and Clinical Features

Patients affected by SBC and treated with CIRT between 2013 and 2016 at the National Center of Oncological Hadrontherapy (CNAO, Pavia, Italy) were retrospectively selected. Inclusion criteria were: (i) prescribed biological dose of 70.4 Gy(RBE) delivered in 16 fractions, (ii) the availability of clinical follow-up at least at three months, and (iii) availability of pre-treatment T1-weighted and T2-weighted (2D, contrast-free) MRI, planning CT and planned biological dose maps. Plans were optimized with a commercial treatment planning system (Syngo RT Planning VC13, Siemens) using a pencil beam algorithm for physical dose calculation and the local effect model (α/β = 2 Gy) for computing the 3D relative biological effectiveness (RBE) [45]. Patients being re-irradiated, or with a different prescribed dose or with different MR acquisition parameters (supplementary materials, Table S1) were excluded from the study. All patients underwent surgery prior to CIRT (data not available for two out of 57 patients). The study was approved by the ethical committee at the Istituto di Ricovero e Cura a Carattere Scientifico (IRCCS) Policlinico San Matteo (id: 20200053536) and informed consent was obtained. 

The LC was chosen as the clinical endpoint and was calculated from the last day of therapy to the date of event or censoring. Recurrence or disease progression in the target volume (adverse LC) was clinically assessed on radiological imaging at follow-up, and was considered an event for survival analysis, whereas progression-free evaluation (favourable LC) referred to censored data. Clinical features consisted of age at the time of treatment, gender, GTV, anatomical location [29], biopsy-proven histology, brainstem and optic pathway involvement, as recorded in the clinical practice (Table 1). 

### 4.2. Data Preparation

**Figure 3:**
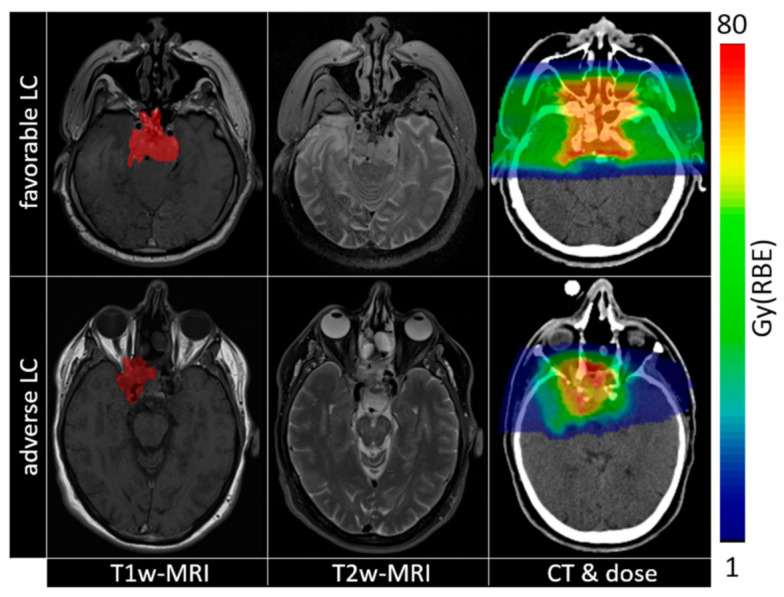
Imaging (from left to right: T1w-MRI, T2w-MRI, CT) and dose maps (overlaid to CT) for patients with opposite local control evaluation (top row favourable LC, bottom row adverse LC). Tumour contours are shown as red overlays on T1w-MRI images.

Within the clinical planning procedure, gross tumour volumes (GTVs) were manually delineated for treatment planning on the planning CT, with the support of a fused MRI and following institutional guidelines. Since manual MRI contours were not available and T1w- and a T2w-MRI were acquired on the same day of the planning CT, GTV contours were conveyed to both T1w- and T2w-MRI through a rigid registration with CT imaging (Figure 3). Before feature extraction, T1w- and T2w-MRI underwent bias field correction [46] and intensity normalization, based on a histogram matching algorithm [47,48]. No denoising strategy was applied because of the undefined noise characteristics of the employed MR sequences. Pre-processing steps were neither applied to CT images (expressed in Hounsfield Units—HU) nor to biological dose maps.

### 4.3. Feature Extraction and Selection

Features were extracted using the open-source software pyradiomics (v2.2.0) [49], which complies with recommendations from the Image Biomarker Standardisation Initiative [50]. Shape features (*n* = 14) were computed from the GTVs segmented on the CT, whereas first-order (*n* = 18) and textural features (*n* = 75) were computed for every modality separately (CT, T1w-MRI, T2w-MRI, dose maps). Textural features described spatial intensity patterns from gray level co-occurrence (GLCM), gray level run length (GLRLM), gray level size zone (GLSZM), gray level dependence (GLDM), and neighbouring gray tone difference (NGTDM) matrices. For all the modalities, features were extracted from the GTV. Details on the feature extraction routines employed are provided in the supplementary materials (supplementary materials, Section S1, Table S2). 

Different methods for dimensionality reduction were tested to mitigate the unbalance between number of features and sample size [51] and to investigate their interplay with the employed models [52]. Ten feature selection routines were applied to radiomic and dosiomic features [53] in a two-step procedure: at first, combinations of unsupervised methods (i.e., based on correlation, clustering, and principal component analysis—PCA) were applied repeatedly and features were then selected based on frequency. Additional details on feature selection routines are reported in the supplementary materials (supplementary materials, Section S2).

### 4.4. Survival Models

A machine learning survival model based on linear survival support vector machines (s-SVM) [54] was adopted and compared to a conventional Cox proportional hazards model regularized with an elastic net penalty (r-Cox, scikit-survival, v. 0.11) [55]. To highlight the potential clinical application of the proposed models, Kaplan-Meier survival curves were finally estimated (lifelines, v. 0.24.3, [56]) for low- and high-risk groups, defined according to the models’ output (Section 4.5 for details).

### 4.5. Experiments

**Figure 4:**
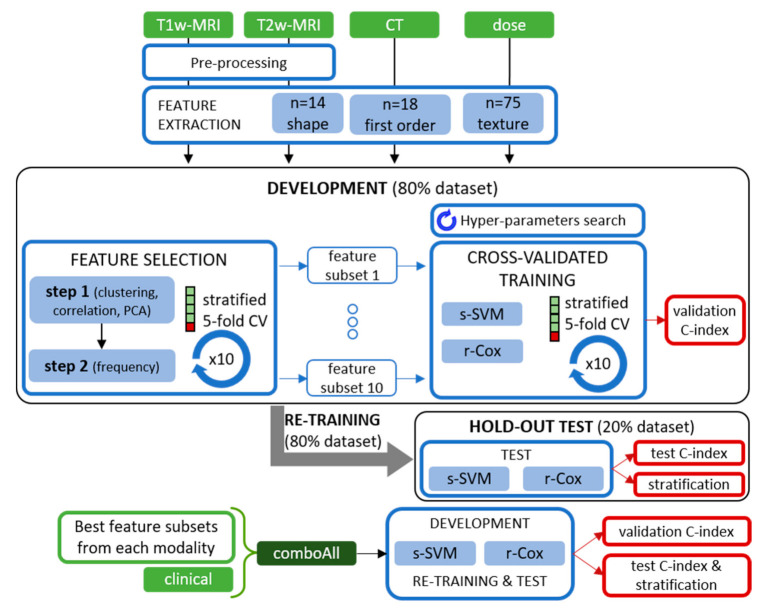
The proposed workflow. Green boxes highlight feature sources, blue boxes represent computational steps and red boxes results. Pre-processing steps are detailed in the supplementary materials, Section S1. A stratified five-fold cross-validation (5-CV) routine was repeated ten times and applied to the training set, for both feature selection and model training during the development phase. The hold-out test set was used to test the re-trained models in terms of models’ performance (test C-index) and ability to stratify patients in different survival curves according to their risk of undergoing an adverse LC event.

Before feature selection, 80% of the patients (*n* = 45) were assigned to the development set, to evaluate the model building procedure, and 20% (*n* = 12) to the hold-out test set, to evaluate the models on totally unseen data (Figure 4). Data was split randomly but ensuring that the proportion of samples associated to adverse and favourable LC was equal. Before models’ training, features were normalized (z-score for s-SVM and L2-norm for r-Cox) and the normalization parameters applied to the unseen data (i.e., validation fold, hold-out data) both for the development and test routines.

During models’ development, a five-fold cross-validation routine was defined so that, in each fold (*n* = 9), various follow-up time durations were present. Folds were created 10 times, each time with a different data split (repeated stratified five-fold CV). During the development phase, feature selection was performed, as reported in Section 4.3. After that the features’ subset was chosen, models’ hyper-parameters were defined, through a grid search, as the combination of parameters that maximized models’ performance (supplementary materials, Section S3, Table S3). The models’ predictive performance was evaluated in terms of the C-index, a generalization of the area under the receiver operating characteristic curve for censored data [57]. Specifically, the median value of the C-indices computed from the validation fold (validation C-index) was chosen as the summarizing metric. Clinical features underwent the same routine, except for the feature selection step, which was not performed.

Each modality (T1w-MRI, T2w-MRI, CT, dose, clinical) was evaluated separately. Then, the single-modality signatures that were associated to the best validation C-index, within each modality, were retained; they were combined into a multi-parametric feature set, to which clinical features were added (comboAll); and the development procedure was repeated (supplementary materials, Section S4).

Subsequently, the single-modality and comboAll models with the highest validation C-index were tested on the hold-out dataset. Since the cross-validated development phase did not provide a unique model as output, models were re-trained on the whole development set and tested on the hold-out dataset. This allowed evaluating the procedure on totally unseen data as, in the development stage, data used to evaluate the models in the validation folds had been previously used to select features and to optimize models’ hyper-parameters. In this phase, models were evaluated in terms of C-index computed over the hold-out testset (test C-index). 

As for evaluating the clinical applicability of the proposed procedure, the stratification cut-off was set to be the median value of the model’s output in the re-training set, and it was applied to both re-training and test data. The estimated survival curves (i.e., Kaplan-Meier survival curves, Section 4.4.) were compared using log-rank tests, setting the significance at α = 0.05. To account for multiple testing, a Bonferroni correction (*n* = 6) was applied to each model, thus leading to a corrected α = 0.0083.

All calculations were performed in Python 3.6, using functionalities from scikit-learn (v. 0.21.3, [53]).

## 5. Conclusions

Radiomic and dosiomic analyses predicting the risk of adverse LC in SBC treated with CIRT were implemented for the first time, integrating MRI, CT, dose maps, and clinical features. Dosiomic and combined features showed promising results in terms of performance and generalization abilities, but a thorough validation is needed before these models can be applied in the clinical practice. Nevertheless, the reported findings support further investigations on radiomic and dosiomic approaches which may improve the understanding of how CIRT treatment affects LC in SBC.

## Figures and Tables

**Table 1:** Clinical information for the whole dataset, reported as median (range) for continuous variables and occurrences for discrete ones. Anatomical location is coded as upper (1), middle (2) or lower clivus (3), or as a combination of those, as observed with respect to internal anatomical landmarks [29]. GTV—gross tumour volume; LC—local control, n.a.—not available.

**Continuous Variables**		**Median (Range)**
Age (Years)		58 (17–81)
GTV (cm^3^)		14.48 (0.39–194.70)
**Categorical Variables**		**Occurrence**
Gender	Female	22
	Male	35
Histology	Conventional	47
	Chondroid	4
	Dedifferentiated	1
	n.a.	5
Anatomical location	1	6
	2	4
	3	2
	1+2	27
	2+3	6
	1+2+3	11
	n.a.	1
Brainstem involvement	Yes	14
	No	42
	n.a.	1
Optic pathway involvement	Yes	10
	No	46
	n.a.	1
**Outcome**		**Occurrence**
LC	Favorable (censored)	40
	Adverse (adverse event)	17

**Table 2:** Validation concordance indices (validation C-index) from s-SVM and r-Cox models built over various feature subsets, defined by 10 features selection routines (second column, details are given in supplementary materials Table S4), from single modalities (T1w- and T2w-MRI, CT, dose, clinical) and from a combination of those (comboAll). Values are reported as median/interquartile range. Best cases for each modality are marked with ^.

Model	Feature Selection Routine	T1w-MRI	T2w-MRI	CT	Dose	ComboAll	Clinical
s-SVM	Routine n. 1	0.58/0.17	0.50/0.22	0.61/0.24	0.73/0.19	0.69/0.27	
	Routine n. 2	0.58/0.17	0.45/0.24	0.62/0.19	0.74/0.25	0.60/0.20	
	Routine n. 3	0.36/0.21	0.60/0.27	0.77/0.24 ^	0.73/0.22	0.69/0.33	
	Routine n. 4	0.36/0.21	0.64/0.33	0.63/0.24	0.77/0.21	0.69/0.33	
	Routine n. 5	0.60/0.24 ^	0.60/0.25	0.58/0.27	0.67/0.20	0.70/0.24	
	Routine n. 6	0.42/0.22	0.67/0.23 ^	0.68/0.27	0.80/0.24 ^	0.46/0.21	
	Routine n. 7	0.54/0.24	0.63/0.22	0.50/0.24	0.74/0.23	0.58/0.25	
	Routine n. 8	0.56/0.23	0.41/0.18	0.54/0.27	0.23/0.24	0.54/0.25	
	Routine n. 9	0.40/0.18	0.47/0.19	0.55/0.31	0.62/0.30	0.73/0.30 ^	
	Routine n. 10	0.42/0.30	0.41/0.30	0.60/0.35	0.64/0.30	0.55/0.15	
	None						0.69/0.23
r-Cox	Routine n. 1	0.60/0.18	0.60/0.27	0.62/0.35	0.62/0.22	0.63/0.33	
	Routine n. 2	0.60/0.18	0.57/0.27	0.62/0.35	0.59/0.20	0.62/0.30	
	Routine n. 3	0.62/0.28	0.43/0.23	0.64/0.28	0.74/0.20	0.69/0.30	
	Routine n. 4	0.62/0.28	0.57/0.27	0.64/0.28 ^	0.69/0.24	0.69/0.30	
	Routine n. 5	0.64/0.20	0.57/0.32	0.54/0.20	0.72/0.27	0.68/0.33	
	Routine n. 6	0.53/0.38	0.50/0.19	0.54/0.18	0.79/0.26 ^	0.75/0.28 ^	
	Routine n. 7	0.65/0.21	0.50/0.24	0.48/0.25	0.73/0.25	0.57/0.32	
	Routine n. 8	0.65/0.21 ^	0.60/0.30	0.54/0.30	0.73/0.25	0.57/0.62	
	Routine n. 9	0.40/0.29	0.63/0.27 ^	0.53/0.19	0.65/0.22	0.75/0.27 ^	
	Routine n. 10	0.56/0.37	0.59/0.26	0.53/0.24	0.67/0.24	0.75/0.27	
	None						0.64/0.26

**Table 3:** Log-rank tests were applied to statistically describe differences between survival curves for patients at high- and low-risk of meeting an adverse local control for the best cases of each modality (i.e., T1w-MRI, T2w-MRI, CT, dose, clinical) and their combination (comboAll), for both s-SVM and r-Cox models. Cases in which the *p*-value pointed to a statistically significant separation (α = 0.0083) in the re-training set are marked with *, whereas ** marks cases in which significance was found in both re-training and test sets.

Model	T1w-MRI	T2w-MRI	CT	Dose	ComboAll	Clinical
s-SVM	0.273	0.176 *	0.176 *	0.002 **	0.067	0.101 *
r-Cox	0.361	0.067	0.213	0.101	0.101	0.213

## Data Availability

The data presented in this study are available upon reasonable request.

## Supplementary Materials

The following are available online at https://www.mdpi.com/2072-6694/13/2/339/s1. Section S1: Detailed Parameters Description, Section S2: Feature Selection Methods, Section S3: Survival Models, Section S4: Building ComboAll Models, Section S5: Selected Features, Table S1. Acquisition parameters for T1w-MRI, T2w-MRI and CT, Section S6: Additional Results, Table S2. Pre-processing and feature extraction parameters for T1w-MRI, T2w-MRI, CT and dose maps, Table S3. Hyper-parameters found for the best performing s-SVM and r-Cox, for single modality, comboAll and clinical models, Table S4. Validation concordance indices (validation C-index) from s-SVM and r-Cox models built over various feature subsets, Figure S1. Standardized T1w-MRI features for high- and low-risk patients as divided by the best performing s-SVM and r-Cox models, according to the validation C-indices, Figure S2. Standardized T2w-MRI features for high- and low-risk patients as divided by the best performing s-SVM and r-Cox models, according to the validation C-indices, Figure S3. Standardized CT features for high- and low-risk patients as divided by the best performing s-SVM and r-Cox models, according to the validation C-indices, Figure S4. Standardized dose features for high- and low-risk patients as divided by the best performing s-SVM and r-Cox models, according to the validation C-indices, Figure S5. Standardized comboAll features for high- and low-risk patients as divided by the best performing s-SVM and r-Cox models, according to the validation C-indices.
