# Supplementary material for: Radiomics and Dosiomics for Predicting Local Control after Carbon-Ion Radiotherapy in Skull-Base Chordoma
Source: Cancers (Basel). 2021 Jan 18;13(2):339. doi: 10.3390/cancers13020339 (PMC7832399; doi:10.3390/cancers13020339)
Supplement: Supplementary file 1 [file cancers-13-00339-s001.pdf]

cancers

Supplementary Materials: Radiomics and Dosiomics for Predicting Local Control after Carbon-Ion Radiotherapy in Skull-Base Chordoma

Giulia Buizza, Chiara Paganelli, Emma D'Ippolito, Giulia Fontana, Silvia Molinelli, Lorenzo Preda, Giulia Riva, Alberto Iannalfi, Francesca Valvo, Ester Orlandi and Guido Baroni

Section S1: Detailed Parameters Description

Relevant acquisition parameters for T1w-MRI, T2-MRI and CT are shown in Table S1. When the parameter type is not applicable, "--" is used. Dose maps were computed at an isotropic resolution of 2 × 2 × 2 mm, using a Syngo RT Planning (VC13, Siemens, Erlangen, Germany).

Table S1. Acquisition parameters for T1w-MRI, T2w-MRI and CT.

Acquisition	T1w-MRI	T2w-MRI	CT
Spatial resolution range (mm)	0.47 × 0.47 × 3 - 0.97 × 0.97 × 3	0.46 × 0.46 × 3 - 0.78 × 0.78 × 3-5	0.98 × 0.98 × 2
TE/TR range (ms)	2.48–11 / 377–887	76–104 / 2400–10951	--
Flip angle range (deg)	67–150	80–150	--
kVp	--	--	120
mAs	--	--	320
Machine (Siemens)	Magnetom Verio		Sensation Open

Before feature extraction, T1w- and T2w-MRI underwent bias-field correction and intensity normalization (Table S2). Additionally, spatial resampling was performed so that the extracted features would refer to the same scale. Dimensionality and resolution at which features were computed are detailed in Table S2. No filter (e.g., wavelet, Laplacian of Gaussians, etc.) was applied prior extraction of the following features [49]:

- Shape (3D, from planning gross tumour volume contours): elongation, flatness, least axis length, major axis length, maximum 2D diameter column, maximum 2D diameter row, maximum 2D diameter slice, maximum 3D diameter, mesh volume, minor axis length, sphericity, surface area, surface volume ratio, voxel volume.
- First-order (from every single-modality imaging): 10th percentile, 90th percentile, energy, entropy, interquartile range, kurtosis, maximum, mean absolute deviation, mean, median, minimum, range, robust mean absolute deviation, root mean squared, skewness, total energy, uniformity, variance.
- Texture:
  - GLCM (Grey Level Co-occurrence Matrix): autocorrelation, cluster prominence, cluster shade, cluster tendency, contrast, correlation, difference average, difference entropy, difference variance, inverse difference, inverse difference moment, inverse difference moment normalized, inverse difference normalized, information measure of correlation 1, information measure of correlation 2, inverse variance, joint average, joint entropy, joint energy, maximal correlation coefficient, maximum probability, sum average, sum entropy, sum squares;
  - GLRLM (Grey Level Run Length Matrix): GL non uniformity, GL non-uniformity normalized, GL variance, high GL run emphasis, long-run emphasis, long-run high GL emphasis, long run low GL emphasis, low GL emphasis, run entropy, run length

non-uniformity, run length non-uniformity normalized, run percentage, run variance, short run emphasis, short run GL emphasis, short run low GL emphasis;
- GLSZM (Gray Level Size Zone Matrix): GL non uniformity, GL non uniformity normalized, GL variance, high GL zone emphasis, large area emphasis, large area high GL emphasis, large area low GL emphasis, low GL zone emphasis, size zone non uniformity, size zone non uniformity normalized, small area emphasis, small area high GL emphasis, small area low GL emphasis, zone entropy, zone percentage, zone variance;
- GLDM (Gray Level Dependence Matrix): dependence entropy, dependence non uniformity, dependence non uniformity normalized, dependence variance, GL non uniformity, GL variance, high GL emphasis, large dependence emphasis, large dependence high GL emphasis, large dependence low GL emphasis, low GL emphasis, small dependence emphasis, small dependence high GL emphasis, small dependence low GL emphasis;
- NGTDM (Neighbouring Gray Tone Difference Matrix): busyness, coarseness, complexity, contrast, strength.

Table S2. Pre-processing and feature extraction parameters for T1w-MRI, T2w-MRI, CT and dose maps.

Processing Step	T1w-MRI	T2w-MRI	CT	Dose
Bias-field correction	N4ITK [46]	N4ITK [46]	none	none
Intensity normalization	Histogram matching [47,48]	Histogram matching [47,48]	none	none
Dimensionality	2D	2D	2D	3D
Resampling	B-Spline	B-Spline	none	none
Resolution / mm	0.65 × 0.65 × 3	0.651 × 0.651 × 3	0.976 × 0.976 × 2	2 × 2 × 2
Bin width for discretization	5	5	50	2

Section S2: Feature Selection Methods

Feature selection was implemented as a two-step routine. In the first step, data was divided for cross-validation in five folds, stratified for follow-up times (stratified five-fold CV), and features were selected by combinations of unsupervised methods (based on correlation, principal component analysis—PCA—or clustering), namely:

- Selecting the features closest to the centroids of a k-means algorithm, where k was chosen through silhouette analysis and k was constrained between 5 and 20 (clustering).
- Choosing an uncorrelated subset of features with size closest to the desired final subset size (correlation);
- Ranking features according to the coefficients from a principal component analysis associated to each of them (PCA);
- A combination of clustering or correlation with PCA ranking, to try to reduce the number of features with respect to the observations (patients) before PCA.

In the second step, after repeating the stratified five-fold CV ten times (10× stratified five-fold CV), each time with a different random data split, features were retained according to the frequency with which they were chosen, following two criteria. These criteria were to either select the ten most frequently chosen features (almost five patients for each feature) or to keep those that had been chosen in more than 20% of the cases, regardless their number (marked as reduced in Table S4). With this second step, features that showed the highest repeatability under data perturbation were retained.

The name of the feature selection routine presented in the tables throughout the manuscript is given by the names of the method used at each step separated by an underscore (e.g., correlation_PCA_reduced).

Section S3: Survival Models

The models evaluated in this work are survival support vector machine (s-SVM [54]) and Cox's proportional hazard model regularized with an elastic net penalty (r-Cox, scikit-survival, v. 0.11 [55]).

A five-fold cross-validated grid search (GridSearchCV, scikit-learn, v. 0.21.3) was set to find the hyper-parameters (Table S3) associated to the highest predictive performance, in terms of the cross-validated concordance index (validation C-index) [57].

Table S3. Hyper-parameters found for the best performing s-SVM and r-Cox, for single modality, comboAll and clinical models.

Modality	s-SVM (α, Optimizer, r)	r-Cox (l1-Ratio)
T1w-MRI	(0.1, direct-count, 1)	0.8
T2w-MRI	(0.01, avltree, 1)	1.0
CT	(0.1, rbtree, 1)	0.8
Dose	(0.001, avltree, 1)	0.8
Clinical	(1, avltree, 1)	1.0
ComboAll	(1e-4, avltree, 1) – correlation_reduced	1e-4 - correlation_reduced<br>1e-5 - PCA

For s-SVM, the hyper-parameters and the respective search grids were:

- alpha (α), searched over [0.0001, 0.001, 0.01, 0.1, 0.5, 1, 10, 100]: it represents the strength of the regularization that should be applied;
- optimizer type, searched among {'avltree', 'rbtree', 'direct-count'}: it refers to the type of optimizer;
- rank ratio (r), searched over [1×10^{-5}, 0.0001, 0.001, 0.2, 0.5, 0.8, 1]: it represents the trade-off between the regression and the raking objective that the s-SVM maximizes. A value of 1 forces the s-SVM to only account for ranking and ignore regression.

For r-Cox, the l1-ratio, which represents the trade-off between L1 and L2 penalization within the elastic net score, was searched over [0.00001, 0.0001, 0.001, 0.01, 0.2, 0.5, 0.8, 1]. An L1-ratio of 0 forces the regularization to follow the L2 paradigm, whereas a value of 1 the L1 paradigm. The model evaluates, during training, different constant values to scale the loss function and optimize the step size (100 weightings were used). The last weight that was employed along the optimization path is used at the prediction stage.

Section S4: Building ComboAll Models

After evaluating single-modality and clinical signatures, these were combined to feed comboAll features to s-SVM and r-Cox, respectively. The two model types were kept completely separated along the process.

Specifically, single-modality signatures associated to the best-performing (validation C-indices) s-SVM, within each modality, were firstly merged with clinical features. Then, feature selection routines were applied, s-SVM hyper-parameters were tuned, and s-SVM models evaluated. The same procedure was applied to single-modality features associated to the best r-Cox in the development set, which were merged with clinical features, selected, exploited for hyper-parameter tuning, and finally fed to r-Cox models.

After these steps were applied, best comboAll cases could be evaluated for s-SVM and r-Cox.

Section S5: Selected Features

Among all the evaluated signatures, those associated to best validation C-index values are shown in Figures S1–S5, for single and combined modalities. Dosiomic features seem to assign higher heterogeneity to patients from the high-risk group with respect to those belonging to the low-risk group, as stratified by the corresponding survival model.

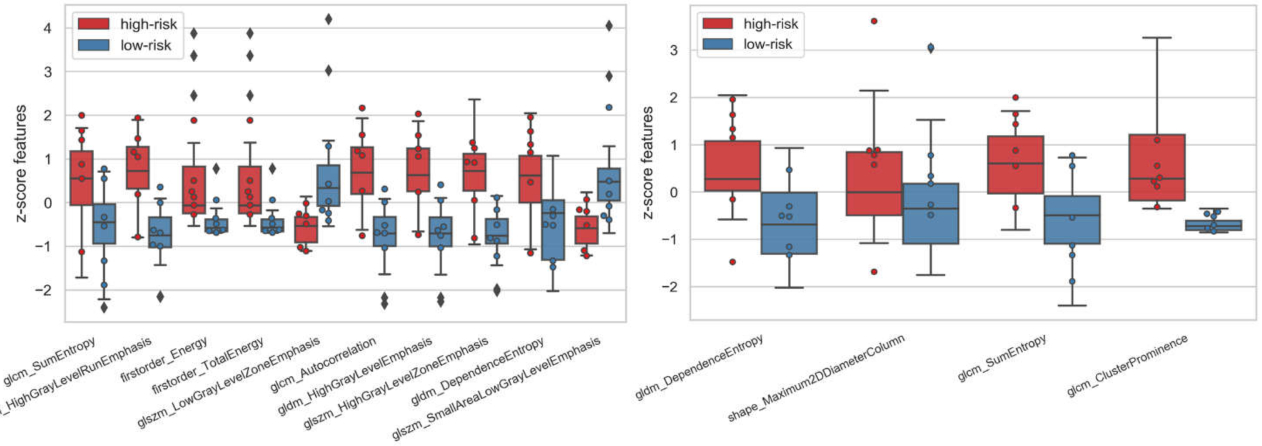

Figure S1. Standardized T1w-MRI features for high- (red) and low-risk (blue) patients as divided by the best performing s-SVM (left) and r-Cox (right) models, according to the validation C-indices. Each model was re-trained on the whole training set (80% dataset), from which the stratification cut-off was estimated, and tested on the hold-out test set (20% dataset). Boxplots show how re-training data was grouped, whereas the overlaid points how test data was divided for high- and low-risk patients.

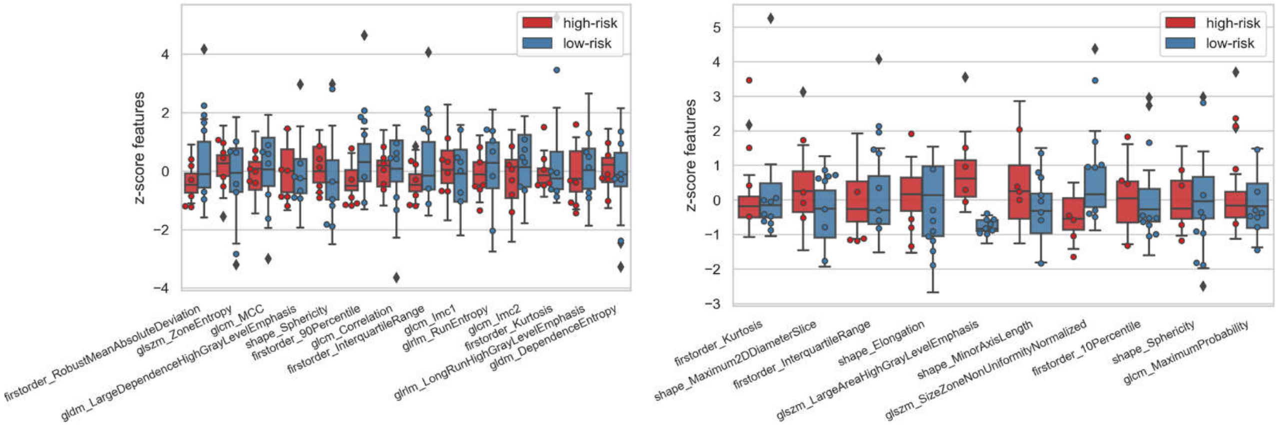

Figure S2. Standardized T2w-MRI features for high- (red) and low-risk (blue) patients as divided by the best performing s-SVM (left) and r-Cox (right) models, according to the validation C-indices. Each model was re-trained on the whole training set (80% dataset), from which the stratification cut-off was estimated, and tested on the hold-out test set (20% dataset). Boxplots show how re-training data was grouped, whereas the overlaid points how test data was divided for high- and low-risk patients.

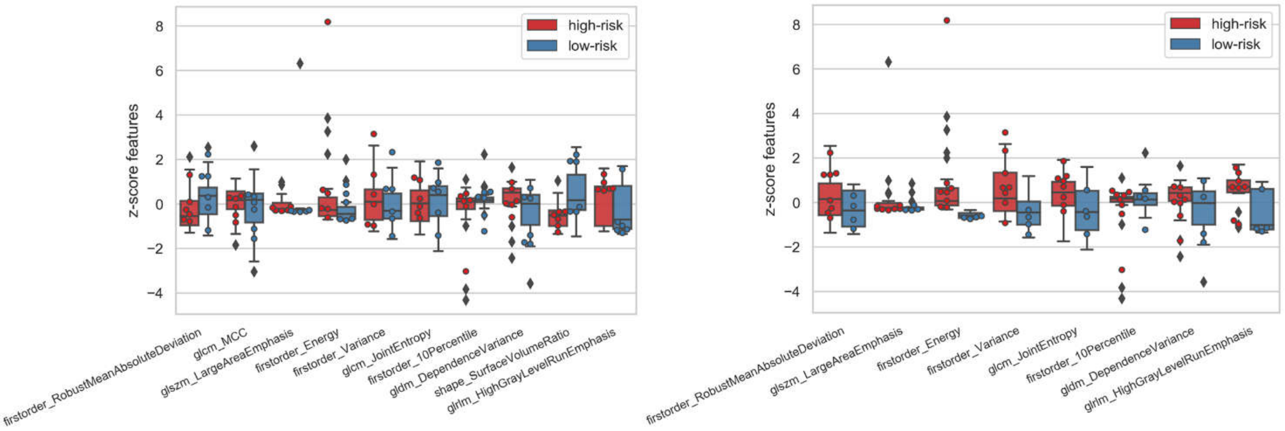

Figure S3. Standardized CT features for high- (red) and low-risk (blue) patients as divided by the best performing s-SVM (left) and r-Cox (right) models, according to the validation C-indices. Each model was re-trained on the whole training set

(80% dataset), from which the stratification cut-off was estimated, and tested on the hold-out test set (20% dataset). Boxplots show how re-training data was grouped, whereas the overlaid points how test data was divided for high- and low-risk patients.

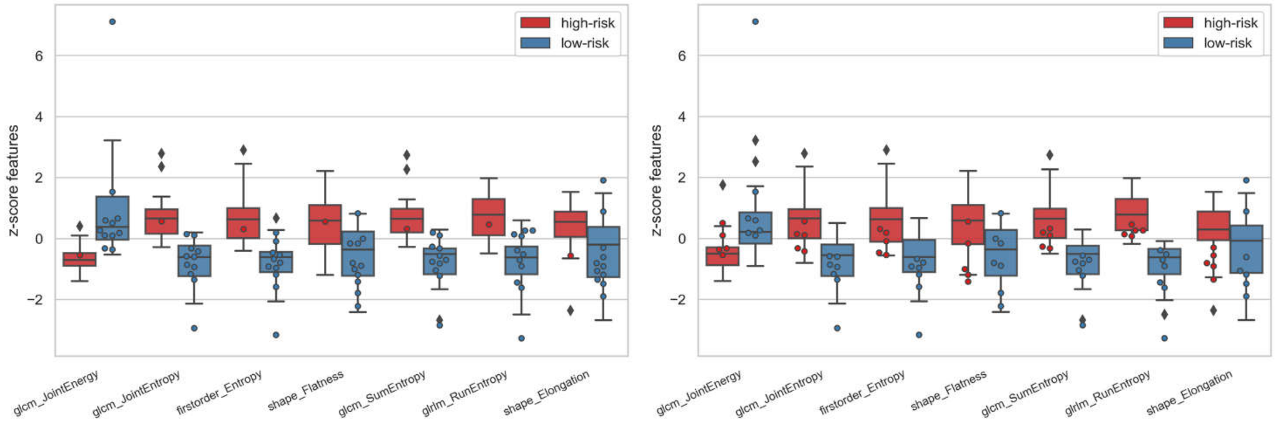

Figure S4. Standardized dose features for high- (red) and low-risk (blue) patients as divided by the best performing s-SVM (left) and r-Cox (right) models, according to the validation C-indices. Each model was re-trained on the whole training set (80% dataset), from which the stratification cut-off was estimated, and tested on the hold-out test set (20% dataset). Boxplots show how re-training data was grouped, whereas the overlaid points how test data was divided for high- and low-risk patients.

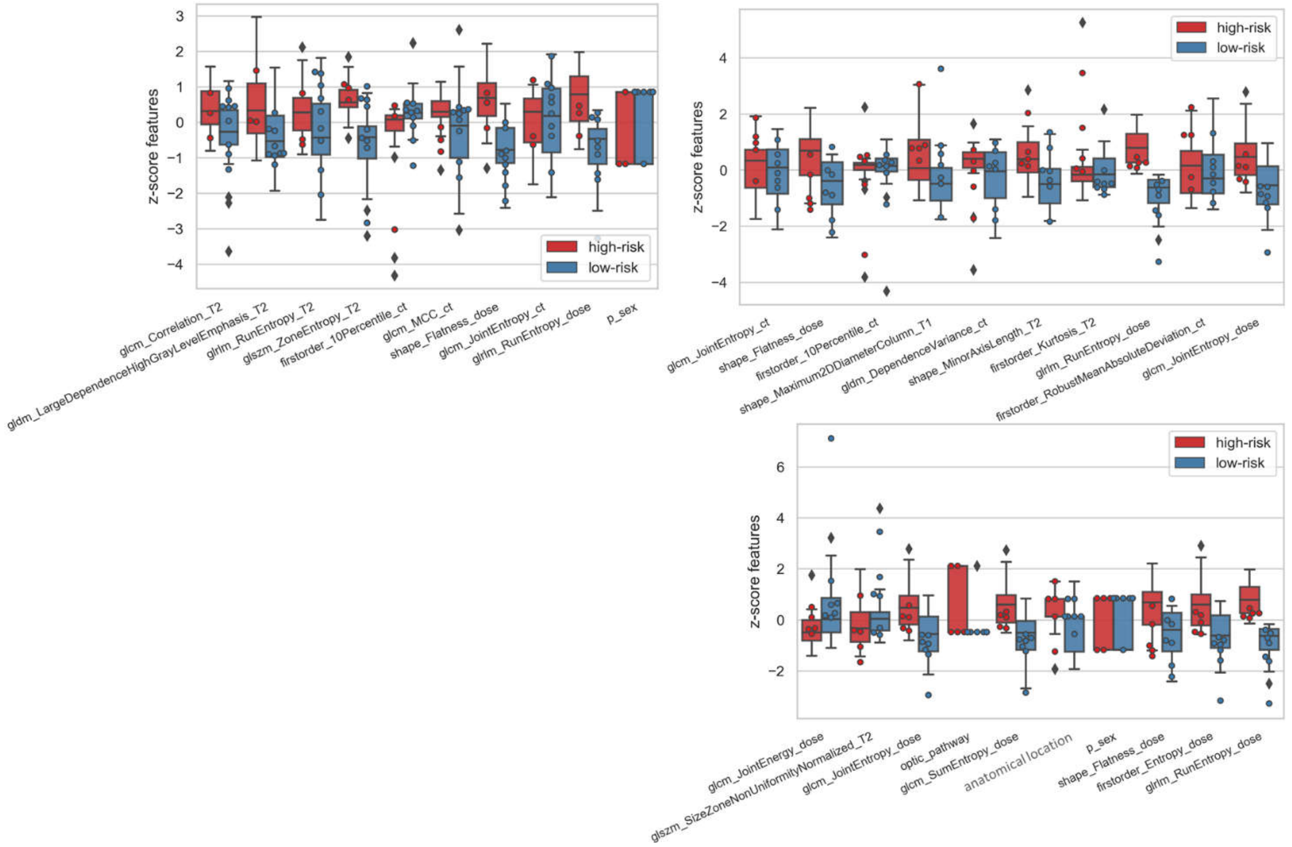

Figure S5. Standardized comboAll features for high- (red) and low-risk (blue) patients as divided by the best performing s-SVM (left) and r-Cox (right) models, according to the validation C-indices. The features in the top row are associated to the PCA-based feature selection routine, whereas the ones in the bottom row with the correlation-based one. The model was re-trained on the whole training set (80% dataset), from which the stratification cut-off was estimated, and tested on the hold-out test set (20% dataset). Boxplots show how re-training data was grouped, whereas the overlaid points how test data was divided for high- and low-risk patients.

Section S6: Additional Results

Results for s-SVM and r-Cox models in the development set are reported as in Table 2, by detailing the name of each feature selection routine (Table S4).

Table S4. Validation concordance indices (validation C-index) from s-SVM and r-Cox models built over various feature subsets, defined by ten features selection routines (second column), from single modalities (T1w- and T2w-MRI, CT, dose, clinical) and from a combination of those (comboAll) are reported as median/interquartile range. Best cases for each modality are marked with ^, whereas cases marked with -- are the same as the line above, since the subset of selected features was the same.

Model	Feature Selection Routine	T1w-MRI	T2w-MRI	CT	Dose	ComboAll	Clinical
s-SVM	Clustering	0.58/0.17	0.50/0.22	0.61/0.24	0.73/0.19	0.69/0.27	
	Clustering_reduced	--	0.45/0.24	0.62/0.19	0.74/0.25	0.60/0.20	
	Clustering_PCA	0.36/0.21	0.60/0.27	0.77/0.24^	0.73/0.22	0.69/0.33	
	Clustering_PCA_reduced	--	0.64/0.33	0.63/0.24	0.77/0.21	--	
	Correlation	0.60/0.24^	0.60/0.25	0.58/0.27	0.67/0.20	0.70/0.24	
	Correlation_reduced	0.42/0.22	0.67/0.23^	0.68/0.27	0.80/0.24^	0.46/0.21	
	Correlation_PCA	0.54/0.24	0.63/0.22	0.50/0.24	0.74/0.23	0.58/0.25	
	Correlation_PCA_reduced	0.56/0.23	0.41/0.18	0.54/0.27	0.23/0.24	0.54/0.25	
	PCA	0.40/0.18	0.47/0.19	0.55/0.31	0.62/0.30	0.73/0.30^	
	PCA_reduced	0.42/0.30	0.41/0.30	0.60/0.35	0.64/0.30	0.55/0.15	
	None						0.69/0.23
r-Cox	Clustering	0.60/0.18	0.60/0.27	0.62/0.35	0.62/0.22	0.63/0.33	
	Clustering_reduced	--	0.57/0.27	0.62/0.35	0.59/0.20	0.62/0.30	
	Clustering_PCA	0.62/0.28	0.43/0.23	0.64/0.28	0.74/0.20	0.69/0.30	
	Clustering_PCA_reduced	--	0.57/0.27	0.64/0.28^	0.69/0.24	0.69/0.30	
	Correlation	0.64/0.20	0.57/0.32	0.54/0.20	0.72/0.27	0.68/0.33	
	Correlation_reduced	0.53/0.38	0.50/0.19	0.54/0.18	0.79/0.26^	0.75/0.28^	
	Correlation_PCA	0.65/0.21	0.50/0.24	0.48/0.25	0.73/0.25	0.57/0.32	
	Correlation_PCA_reduced	0.65/0.21^	0.60/0.30	0.54/0.30	0.73/0.25	0.57/0.62	
	PCA	0.40/0.29	0.63/0.27^	0.53/0.19	0.65/0.22	0.75/0.27^	
	PCA_reduced	0.56/0.37	0.59/0.26	0.53/0.24	0.67/0.24	--	
	None						0.64/0.26

For the cases in which the validation C-index was the highest (marked with ^ in Table S4), models were evaluated in the ability to significantly stratify low- and high-risk patients (Table 3) and their performance was evaluated in terms of test C-index (Table S5).

Table S5. Test concordance indices (test C-index) from s-SVM and r-Cox models built from single modalities (T1w- and T2w-MRI, CT, dose, clinical) and from a combination of those (comboAll) are reported.

Model	T1w-MRI	T2w-MRI	CT	Dose	ComboAll	Clinical
s-SVM	0.64	0.91	1.00	1.00	1.00	1.00
r-Cox	0.36	1.00	1.00	1.00	1.00	1.00
